# Is There a Relationship Between Physical Performance Factors and Adverse Reactions to Foodstuffs? The ALASKA Study

**DOI:** 10.3390/nu16244384

**Published:** 2024-12-20

**Authors:** Lisset Pantoja-Arévalo, Eva Gesteiro, Margarita Pérez-Ruiz, Songxin Tang, Rafael Urrialde, Marcela González-Gross

**Affiliations:** 1 ImFINE Research Group, Department of Health and Human Performance, Universidad Politécnica de Madrid, 28040 Madrid, Spain; eva.gesteiro@upm.es (E.G.); margarita.perez@upm.es (M.P.-R.); songxin.tang@alumnos.upm.es (S.T.); marcela.gonzalez.gross@upm.es (M.G.-G.); 2 Department of Genetics, Physiology and Microbiology, Universidad Complutense de Madrid, 28040 Madrid, Spain; rurriald@ucm.es; 3 Department of Pharmaceutical and Health Sciences, Universidad CEU San Pablo, 28003 Madrid, Spain; 4 Department of Nutrition, Universidad de Valladolid, 47002 Valladolid, Spain; 5 Biomedical Research Center of Pathophysiology of Obesity and Nutrition-CIBERobn, Carlos III Health Institute, 28029 Madrid, Spain

**Keywords:** cardiorespiratory fitness, exercise test, food hypersensitivity, immunoglobulin E, immunoglobulin G, physical fitness, physical functional performance

## Abstract

**Background/Objectives:** An optimal physical condition has beneficial effects in adults at risk of chronic diseases. However, research data on how adverse reactions to food (ARFSs) are linked to physical performance are lacking. The aims of this study were (a) to investigate the prevalence of ARFS according to age; (b) to analyze physical performance level according to the type of ARFS; and (c) to determine the probability of having a positive ARFS according to physical performance levels. **Methods:** A cross-sectional study with 254 Spanish adults (61% women; mean age 43.7 ± 13 y) scoring ≥ 6 in PSIMP-ARFSQ-10 (pathologies and symptomatology questionnaire associated with adverse reactions to foodstuffs) was conducted in the region of Madrid, Spain, following the ALASKA study protocol. Immune-mediated variables used to measure ARFS were sIgE and sIgG_4_ antibody reactions (AbR) (type 1 and type 2 food hypersensitivities, respectively); non-immune-mediated variables used to measure ARFS were lactose intolerance and fructose malabsorption. Physical performance variables were body balance, leg power, sit-to-stand speed, resting heart rate, handgrip strength, and cardiorespiratory fitness. Statistical significance was set at 0.05. **Results:** The most prevalent sIgE- and sIgG_4_-mediated ARFSs were against legumes (53% and 46%; 60% and 68% in subjects with ≤45 y and >45 y, respectively). Handgrip strength was significantly lower in subjects positive for lactose intolerance compared to subjects negative for lactose intolerance (*p* < 0.05). Both the positive mean sIgE and sIgG_4_ AbR were significantly associated with high physical performance (*p* < 0.05). Subjects with high physical performance showed a 1.5-fold increase in the odds of the positive mean total sIgE and positive sIgG_4_ AbR against legumes. **Conclusions:** In conclusion, subjects aged 45 or younger had a higher prevalence of total type 1 and type 2 food hypersensitivities than subjects older than 45 y. Positive lactose intolerance was linked to lower values of handgrip strength. Subjects with high physical performance, whether male or female, aged ≤45 years, or with a BMI of ≥25, showed significant odds of experiencing type 1 food hypersensitivity to nuts.

## 1. Introduction

Food allergies, together with other related adverse reactions to foodstuffs (ARFSs), are currently considered a global public health concern and are among the five most important chronic diseases of the world according to the World Health Organization due to their increasing prevalence in the past decade [1]. The most recent available European food allergy prevalence data based on food allergen-specific serum IgE (sIgE) antibody reactions (AbRs) were published by the European Academy of Allergy and Clinical Immunology (EAACI) in 2023, reporting a prevalence of 16.6% for food allergies based on sIgE AbR in European individuals, specifically 18.4% and 11.2% for children and adults, respectively [2].

According to the recently published ARFS classification by Pantoja-Arévalo et al. (2024), ARFS refers to any immune-mediated and non-immune-mediated food reaction, such as food allergy or type 1 food hypersensitivity (mostly sIgE-mediated ARFS), and type 2 food hypersensitivity (mostly sIgG_4_-mediated ARFS) refers to immune-mediated reactions, while food malabsorption (e.g., fructose malabsorption) and food intolerance (e.g., lactose intolerance) are referred to as non-immune-mediated food reactions, among other ARFSs [3]. Type 1 and type 2 food hypersensitivities have been linked to the food allergy pathogenesis identification and to self-reported symptoms related to ARFS, respectively [4,5]. Additionally, individuals with lactose intolerance and fructose malabsorption presenting symptoms related to the digestive system have been observed to have higher physical commotions than those without lactose intolerance [6].

ARFSs encompass a range of related aspects that can be affected by intrinsic and extrinsic factors [3]. Optimal cardiorespiratory fitness and other physical performance variables have been shown to have a positive apparent impact on the health of adults with chronic diseases in developed countries. However, individuals presenting better physical performance may be exposed to the opposite probability when evaluating food hypersensitivity. For instance, for athletes with immune-mediated allergies to environmental allergens, such as pollen, exercise may exacerbate their physical and digestive conditions due to increased ventilation during exercise [7]. Similarly, for individuals without any related diagnosed ARFS disease but presenting related symptomatology, exercise may still worsen their physical and digestive performance due to increased exercise-related metabolic inflammation, especially in the adipose tissue [8]. Cardiorespiratory fitness, strength, speed, balance, and heart rate have been identified as potential associated physical performance factors in individuals presenting ARFS-related symptomatology, specifically when exercise is presumed to alter gut absorption [9,10]. Nevertheless, there is a lack of robust data regarding the direct relationship between ARFS and physical performance-related variables.

The mechanisms of food allergy may vary among different populations worldwide. It has been observed that individuals experience variations in the positivity of ARFS when exposed to the same amount or even type of food allergen at different stages of life [11]. Attempts have been made to explain these variations describing several general associated factors in ARFSs, such as family history, dietary habits, environmental and food allergen exposure, and other related aspects [12]. Studying ARFSs in the physical activity and sport sciences context is essential due to the “dual allergen exposure hypothesis”, which suggests that changes in environmental conditions during sports can contribute to food-related ARFS disease progression [13]. Therefore, for the present study, it is considered that factors such as, sex, age, body mass index (BMI), specific symptomatology, cardiorespiratory fitness, and other physical performance variables could help in comprehending the nature of the ARFS in this scenario. Consequently, understanding the possible factors related to ARFSs would significantly help improve ARFS management, intervention, and treatment studies in the future.

The aims of this study were (a) to investigate the prevalence of adverse reactions to foodstuffs according to age; (b) to analyze the physical performance level according to the type of adverse reaction to foodstuffs; and (c) to determine the probability of having a positive ARFS according to physical performance levels in a sample of Spanish adults.

## 2. Materials and Methods

The methodology of this study was carried out following the published ALASKA study protocol previously described elsewhere [3].

### 2.1. Study Design and Participants

The present study analyzed data collected from the ALASKA study. This was a cross-sectional study conducted from April 2022 to May 2023 in the Region of Madrid, Spain. A total of 295 Spanish adults were eligible to participate. Individuals aged ≥18y, with no medical restrictions on physical activity, those maintaining a regular sleep schedule, those who are not heavy smokers (<25 cigarettes/day), not pregnant, with no prior diagnosis of ARFS, and a PSIMP-ARFSQ-10 score of ≥6 volunteered to participate in this study. Twenty-one participants did not meet the inclusion criteria, ten participants did not sign the consent follow-up section, six participants were excluded for attrition and loss to follow-up, and four participants had missing data. A total sample of 254 Spanish adults with complete and plausible data regarding the assessments in Table 1 were evaluated and considered as a valid sample.

### 2.2. Study Variables

The variables and instruments used in this study are summarized in Table 1. Questionnaires and forms were administered online using the Research Electronic Data Capture Platform (RedCap^®^), hosted in the Supercomputing and Visualization Center of the Madrid Community of the Universidad Politécnica de Madrid (CESVIMA-UPM). Diseases, signs, and symptoms related to any type of ARFS were assessed using the validated PSIMP-ARFSQ-10 tool [14]. Participants supplied information on their date of birth, age, sex, level of education, employment situation, occupation, living situation (number of people living together) and home city using the demographics and clinical characteristics form published in the ALASKA study protocol [3].

Subjects were organized by age groups into a younger age group (≤45 y) and older age group of adults (>45 y) according to previous observations in which noticeable changes on IgE AbR prevalence were manifested [21,22,23]. BMI was evaluated according to the classification used by the National Institute of Health and the World Health Organization [16]. Physical activity was arranged in accordance with the World Health Organization 2020 guidelines on physical activity and sedentary behavior [24]. ARFS were categorized based on the ALASKA study protocol classification into immune-mediated reactions (type 1 and type 2 hypersensitivities) and non-immune-mediated reactions (lactose intolerance and fructose malabsorption) [3]. Each ARFS subcategory was also evaluated as sIgE-only, sIgG_4_-only, lactose intolerance-only and fructose malabsorption-only, indicating individuals with just a single positive subcategory of either immune- or non-immune-mediated reactions. Cardiorespiratory fitness levels were classified according to 95% of the estimated good age–sex VO_2_max value below which there is risk of developing major health problems (mainly cardiovascular diseases, diabetes, and/or hypertension) [25]. Moreover, maximal isometric handgrip strength was recorded after at least two attempts from each hand. The handle width of the dynamometer was adjusted to subjects’ hand sizes, and subjects were instructed to maintain a neutral standing position while encouraged to squeeze the dynamometer and exert maximum force.

### 2.3. Statistical Analysis

Descriptive statistics consisted of the mean and standard deviation (SD) for continuous variables, as well as frequency and percentage for categorical variables. Kolmogorov–Smirnov tests were used to check for the normality of the continuous data.

Based on previous findings [4,14] where a total raw PSIMP-ARFSQ-10 score of 6 was determined to be related to ARFS, PSIMP-ARFSQ-10 scores were calculated as raw scores, meaning a raw punctuation value of 1 for each single item of the PSIMP-ARFSQ-10 questionnaire. Following the standard practice in scoring questionnaires [26], a raw punctuation value of 1 was assigned to the PSIMP-ARFSQ-10 questionnaire items, followed by the sums of these raw scores by sections (each section included the assigned item weight during the validation of the questionnaire [14]), and the sums were calculated for reliable comparisons.

A model utilizing linear regression and hierarchical multiple regression analysis was used to create different regression equations predicting the association between sex, age, living situation, physical activity, BMI, symptomatology, and physical performance variables (VO_2_max values, maximal handgrip strength, leg power, sit-to-stand speed, body balance, and resting heart rate). Age–sex adjustments were applied to all physical performance variables, and median cut off values of maximal handgrip strength and cardiorespiratory fitness were determined to classify the sample into groups with low and high physical performance.

Binary logistic regression was performed using dichotomic datasets of physical performance levels. Odds ratios (ORs) were used as measurement of association and random-effects risk model. Standard procedures of statistics in public health were followed, and three main interpretations for an OR were considered: no associated factor, protective factor, and associated factor with higher odds of ARFSs. If an OR was equal to 1, there was no association between the physical performance level and the odds of ARFSs. If the OR was <1, the physical performance level was considered a protective factor; finally, if the OR was >1, the physical performance level was considered a significant associated factor with higher odds of ARFSs [27,28]. Statistical significance was set at 0.05.

### 2.4. Ethical Considerations

This research was performed in accordance with the Ethical Guidelines of the Declaration of Helsinki of 1964, revised in Fortaleza (2013) [29], and following the Spanish and European regulations on data protection [30]. The protocol was approved by the Ethics Committee of the Universidad Politécnica de Madrid (reference number 20200602) and registered on ClinicalTrials.gov (Clinical Trials ID NCT05802017).

The ALASKA study was carried out by qualified professionals in medical, clinical, food science, nutritional and physical activity, and sport sciences practices. These experts were part of the ImFINE Research Group of the Department of Health and Human Performance of the Faculty of Physical Activity and Sport Sciences of the Universidad Politécnica de Madrid. Signed informed consent forms were obtained from all participants before their participation commencement.

## 3. Results

### 3.1. Sample Characteristics

A total sample of 254 Spanish adults participated in this study (61% women) with a mean age of 43.71 ± 12.61 years. Data regarding demographics and descriptive characteristics of the ALASKA study are presented in Table 2. The median age was 44, the median age for men was 48, and the median age for women was 42. The men were significantly older than the women (Table 3); however, there were no significant differences in sociodemographic characteristics (education level, job, and living situation) between men and women.

More than 60% of the studied sample presented a normal weight with no significant differences between the men’s and women’s categories of BMI. Furthermore, the results in Table 2 show statistically significant anthropometric differences between the men’s and women’s BMI, height, weight, waist circumference, waist-to-hip ratio, and body composition variables (all *p* < 0.05).

The women revealed a significantly higher total PSIMP-ARFSQ-10 score associated with ARFS pathogenesis (*p* < 0.05) and a particularly higher nervous system symptomatology PSIMP-ARFSQ-10 score (*p* < 0.001) than the men (Table 3). The estimated VO_2_max, maximal handgrip strength, and leg power distribution values were significantly higher in men than women (all *p* < 0.001). However, non-significant sex differences were found between other physical performance variables, specifically the sit-to-stand speed and body balance. In contrast, the resting heart rate distribution values were significantly higher in women than men (*p* < 0.05) (Table 3).

The mean ages for the younger and older age groups of adults were 33.77 ± 7.55 y and 54.99 ± 5.90 y, respectively. Both the mean gastrointestinal and nervous system symptomatology PSIMP-ARFSQ-10 scores were higher in the younger age group than the older age group of adults (*p* < 0.05). The gastrointestinal PSIMP-ARFSQ-10 scores were still higher for the younger age group than the older age group of adults who were women (*p* < 0.05). Self-reported physical activity in men was worse in the older than in the younger age group of adults, with twice as many adults of the older age group not meeting the World Health Organization’s recommendations compared to the younger age group (20% vs. 10%). Regarding maximal handgrip strength, there were no significant differences regarding age groups in men, while the younger age group of women showed a higher maximal handgrip strength than the older age one (*p* < 0.05). Leg power and the sit-to-stand speed were significantly higher in the younger group than the older age group of adults in both men and women (both *p* < 0.001). The estimated VO_2_max was significantly higher in the younger age group, maintaining its significance when analyzing the specific cardiorespiratory fitness levels between the younger age group and the older age groups of adults. The proportion of subjects showing good cardiorespiratory fitness was 70% belonging to the young age group and 50% of adults belonging to the older age group (Table 3).

### 3.2. Prevalence of Adverse Reactions to Foodstuffs According to Age Groups

**Figure 1:**
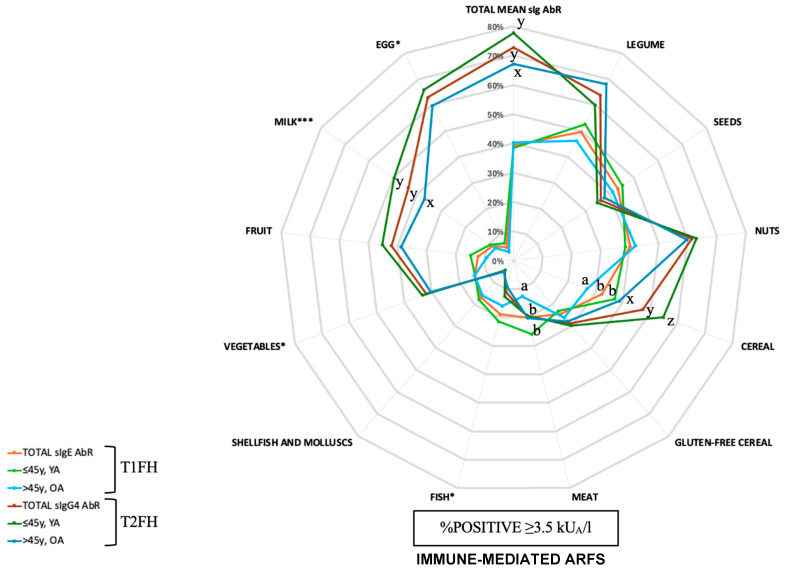
Prevalence of type I and type II hypersensitivity reactions according to age groups. * *p*< 0.05, *** *p*< 0.001. Asterisks “*” beside food groups show statistical differences between overall orange color curves and mean sIgE AbR compared to total mean sIgG_4_ AbR among 12 established food groups. Values bearing different letters are significantly different (a ≠ b ≠ c; x ≠ y ≠ z). kU_A_/L, kilounits of allergen per liter; sIg, serum immunoglobulins; sIgE, serum immunoglobulin E; sIgG_4_, serum immunoglobulin G_4_; T1FH, type 1 food hypersensitivity; T2FH, type 2 food hypersensitivity.

Figure 1 presents the prevalence of positive type 1 and type 2 food hypersensitivities across different age groups. The most prevalent positive sIgE-mediated or type 1 food hypersensitivity and sIgG_4_-mediated or type 2 food hypersensitivity were legumes (50% and 64%), nuts (40% and 61%), and egg (5% and 63%) (*p* < 0.05), respectively. The most prevalent type 1 food hypersensitivities in adults in the younger age group were legumes (53%) and seeds (45%), while they were legumes (46%) and nuts (42%) in adults in the older age group. Analogously, the most prevalent type 2 food hypersensitivities in adults in the younger age group were eggs (66%) and nuts (63%), while they were legumes (68%) and eggs (60%) in adults in the older age group.

#### 3.2.1. Type 1 Food Hypersensitivity

The mean total sIgE AbR was 4.22 ± 4.9 kUA/L, which is almost 40% of sIgE AbR positivity in the studied sample. Nearly half of the subjects showed positive food-specific sIgE AbR against legumes, seeds, and nuts, while nearly one-third presented positive sIgE AbR against cereal and gluten-free cereal (Figure 1). The shellfish food group was the only group showing significant differences by sex, with a higher prevalence of positive sIgE AbR in women compared to men (20.6% vs. 11.1%, respectively) (*p* < 0.05). The mean total food allergen-specific sIgE AbR for the younger and older age groups were 4.6 ± 5.6 and 3.8 ± 3.9 kUA/L, respectively (*p* > 0.05). However, the cereal and meat food groups showed a statistically higher prevalence of positive sIgE AbR in the younger age group compared to the older age group of adults (both *p* < 0.05). When comparing food allergen-specific sIgE AbR by sex between the younger and older age groups, a higher prevalence of positive sIgE AbR against seeds, fish, and meat was found among younger age men compared to older age men, while a higher prevalence of positive sIgE AbR against meat and egg was found among younger age women compared to older age women (all *p* < 0.05).

#### 3.2.2. Type 2 Food Hypersensitivity

The mean total sIgG_4_ AbR was 13.65 ± 13.93 kUA/L, which is around 70% of sIgG_4_ AbR positivity in the studied sample. Approximately two thirds of the subjects showed a positive food-specific sIgG_4_ AbR against legume, nut, and egg-specific sIgG_4_ AbR, followed by around one-third of subjects with positive specific AbR against seeds, cereal, gluten-free cereal, vegetable, fruits and milk (Figure 1). Women showed a higher prevalence of positive sIgG_4_ AbR against the legume food group compared to men (68.4% vs. 56.6%, respectively) (*p* < 0.05), while men showed higher prevalence of positive sIgG_4_ AbR against the gluten-free cereal, seeds, fish, and meat food groups compared to women (39.4% vs. 21.9%, 45.5% vs. 30.3%, 20.2% vs. 5.2%, and 26.3% vs. 15.5%, respectively) (all *p* < 0.05). The mean total food allergen-specific sIgG_4_ AbR values for the younger and older age groups were 14.37 ± 14.12 and 12.84 ± 13.71 kUA/L, respectively (*p* > 0.05). However, a higher prevalence of positive sIgG_4_ AbR values against the cereal and milk food groups was found in the younger age group compared to the older age group (both *p* < 0.05). When comparing food allergen-specific sIgG_4_ AbR by sex between the younger and older age groups, a higher prevalence of positive sIgG_4_ AbR against cereal was found among younger age men compared to older age men (*p* < 0.05). Similarly, a higher prevalence of positive sIgG_4_ AbR against cereal and fish was found among younger age women compared to older age women (all *p* < 0.05). On the other hand, the legume food group showed a higher prevalence of positive sIgG_4_ AbR values in the older age group compared to the younger age group.

Further information about type 1 and type 2 food hypersensitivities by sex and age are referred to in Table S1.

#### 3.2.3. Lactose Intolerance and Fructose Malabsorption

**Figure 2:**
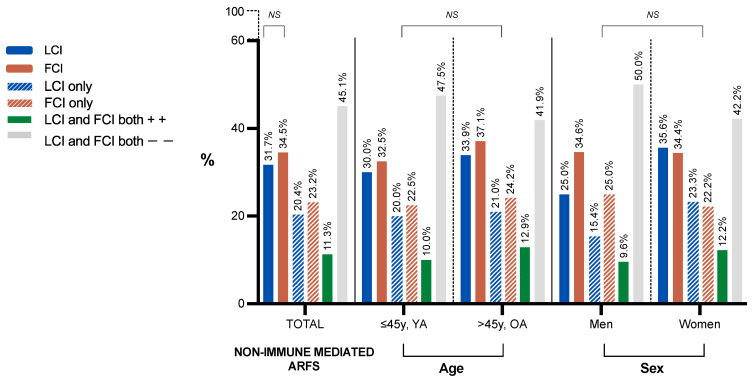
Prevalence of lactose and fructose malabsorption according to age and sex groups. OA, older age group; YA, younger age group; LCI, lactose intolerance; FCI, fructose malabsorption; *NS*, statistically non-significant; y, years of age.

Around one-third of the studied subjects showed either positive lactose or fructose malabsorption, and around one out of ten subjects presented both positive lactose intolerance and fructose malabsorption. The highest prevalence of non-immune-mediated ARFS in the studied sample was found in the older age group, although there were no significant differences among the evaluated age and sex groups (Figure 2).

Table S3 of the Supplementary Material of this manuscript shows ARFS types by sex and age groups. Briefly, the prevalence was 28% for positive immune-mediated ARFSs and 11.3% for positive non-immune-mediated ARFSs. However, while examining the prevalence of immune-mediated ARFSs by the established sex and age groups, women older than 45 y had a greater prevalence of immune-mediated ARFSs compared to women aged 45 or younger (*p* < 0.01). Regarding non-immune-mediated ARFSs, women older than 45 y showed a higher prevalence of positive lactose intolerance than those aged 45 or younger (*p* < 0.05). No further significant differences were found between sex and age groups with positive ARFSs (*p* > 0.05).

### 3.3. Physical Performance

#### 3.3.1. Physical Performance Variables and Sample Characteristics

**Figure 3:**
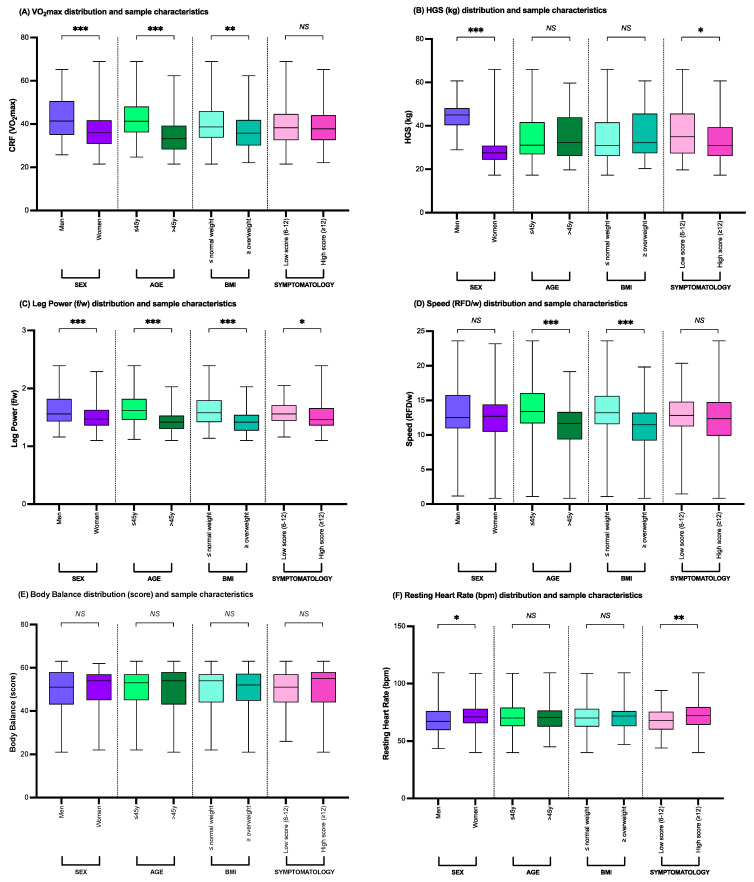
Physical performance variables and sample characteristics. * *p* < 0.05, ** *p* < 0.01, *** *p* < 0.001. ARFS, adverse reaction to foodstuffs; BMI, body mass index; bpm, beats per minute; CRF, cardiorespiratory fitness; f/w, force/weight; HGS, handgrip strength; *NS*, statistically non-significant; RFD/w, rate of force development/weight (sit-to-stand speed); VO_2_max, maximal oxygen consumption; y, years of age.

Significant associations were found between the physical performance variables (cardiorespiratory fitness as estimated VO_2_max, maximal handgrip strength, leg power, sit-to-stand speed, body balance, and resting heart rate distributions) and the studied categories of sex, age, BMI, and symptomatology (Figure 3). There were no significant differences between physical performance variables and self-reported physical activity and between physical performance variables and the subjects’ living situations (number of people living with) (*p* > 0.05). Significant associations, endorsing men, were observed between the estimated VO_2_max, maximal handgrip strength, and leg power with sex categories (all *p* < 0.001). In addition, women exhibited significantly higher resting heart rate values. There were no significant associations between the men’s and women’s sit-to-stand speed and body balance values. The younger age group significantly outperformed the older age group in the cardiorespiratory fitness, leg power, and sit-to-stand speed variables (all *p* < 0.001). Cardiorespiratory fitness and leg power were significantly associated with BMI, and it was observed that greater distribution values of the estimated VO_2_max and leg power were significantly related to BMI values of <25 (*p* < 0.01). Furthermore, greater distribution values of maximal handgrip strength, leg power, and resting heart rate were significantly associated with lower PSIMP-ARFSQ-10 scores (scores from 6 to 12) in the total sample of studied subjects (all *p* < 0.05). Sex, age, BMI, and symptomatology PSIMP-ARFSQ-10 scores were all contributing factors in leg power distribution values (all *p* < 0.05).

#### 3.3.2. Physical Performance Variables and Adverse Reactions to Foodstuffs

**Figure 4:**
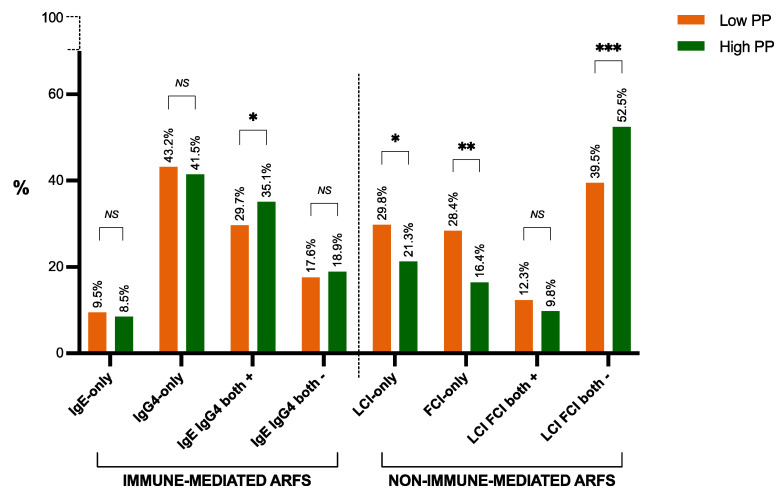
Physical performance according to types of ARFSs. * *p* < 0.05, ** *p* < 0.01, *** *p* < 0.001. ARFS, adverse reactions to foodstuffs; FCI, fructose malabsorption; FCI-only, positive fructose malabsorption and negative lactose intolerance subjects; IgE, immunoglobulin E; IgE-only, positive sIgE AbR and negative sIgG_4_ AbR subjects; IgG_4_, immunoglobulin G_4_; IgG_4_-only, positive sIgG_4_ AbR and negative sIgE AbR subjects; LCI, lactose intolerance; LCI-only, positive lactose intolerance and negative fructose malabsorption subjects; *NS*, statistically non-significant; PP, physical performance.

The prevalence of subjects testing positive for both type 1 and type 2 food hypersensitivities was greater in those with high physical performance compared to those with low physical performance. Meanwhile, subjects with positive lactose intolerance were significantly associated with low physical performance compared to high physical performance (Figure 4). The maximal handgrip strength showed a significant decrease with positive lactose intolerance compared to negative lactose intolerance (*p* < 0.05). Nonetheless, there were no significant variations in cardiorespiratory fitness between the established positive groups of food hypersensitivities and food intolerance. Nevertheless, some associations have been observed while studying the 12 food groups and physical performance variables (Table S2).

Positive food-specific sIgG_4_ AbR was significantly associated with several physical performance variables. Food allergen-specific sIgG_4_ AbR was positively related to higher cardiorespiratory fitness values for the cereal, fish, nut, and shellfish food groups; higher maximal handgrip strength values for the milk and meat food groups; higher sit-to-stand speed values for the legume food group; and higher balance values for the fish food group. In contrast, food-specific sIgG_4_ AbR were related to lower sit-to-stand speed values for the cereal food group and resting heart rate values for the egg food group (all *p* < 0.05). No significant differences in higher leg power or in higher or lower physical performance values for food-specific sIgE AbR were found (Figure S1).

Subjects belonging to the older age group had more significant positive food allergen-specific sIgE and sIgG_4_ AbR values against the studied food groups when higher levels of the analyzed physical performance variables were observed. Subjects older than 45 y showed significant higher values of cardiorespiratory fitness when there were positive sIgG_4_ AbR values against cereal, fish, and seeds or when there were positive sIgE AbR values against eggs. In addition, a higher maximal handgrip strength was found when there were positive sIgG_4_ AbR values against fish or when there were positive sIgE AbR values against meat; moreover, there was higher leg power when there were positive sIgE AbR values against vegetables. Furthermore, a higher sit-to-stand speed was found when there were positive sIgG_4_ AbR values against cereals, fish, and legumes, and finally, there was higher body balance when there were positive sIgG_4_ AbR values against fish, shellfish, and mollusks or when there was positive sIgE AbR values against meat, seeds, shellfish, and mollusks. There were no significant associations between positive food allergen-specific AbR values and resting heart rate (Figure S2).

### 3.4. Physical Performance as an Associated Factor in Adverse Reactions to Foodstuffs

**Figure 5:**
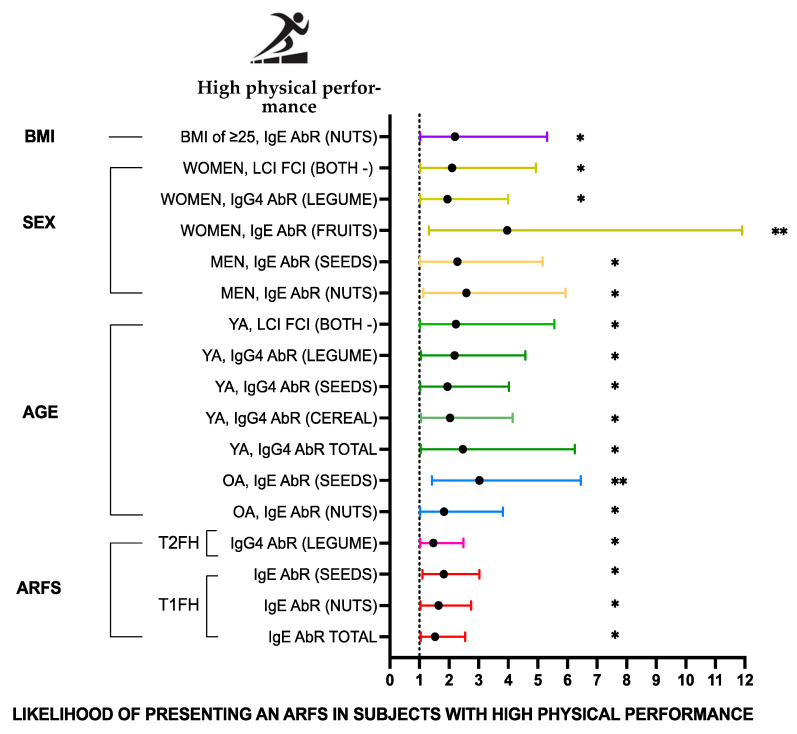
Odds ratio associations between physical performance and ARFS food groups. * *p* < 0.05, ** *p* < 0.01. AbR, antibody reactions; ARFS, adverse reactions to foodstuffs; BMI, body mass index; LCI, lactose intolerance; FCI, fructose malabsorption; OA, older age group (>45 y); OW, overweight; T1FH, type 1 food hypersensitivity; T2FH, type 2 food hypersensitivity; YA, younger age group (≤45 y); both -, both negative.

In order to comprehend the connection between physical performance and ARFSs, logistic regression models were constructed, adjusting the data for demographic factors and sample characteristics (age, sex, and BMI). ORs were determined to measure how strongly having a better physical performance was associated with having any of the analyzed categories of ARFSs (Figure 5).

Subjects with high physical performance were over 1.5 times as likely to have experienced a positive type 1 food hypersensitivity compared to subjects with low physical performance (45.3% vs. 35.1%, respectively; OR = 1.53; 95% CI (1.00, 2.54); *p* < 0.05). Men with high physical performance showed a 2.6-fold increase in the odds of positive sIgE-mediated food hypersensitivity to nuts compared to men with low physical performance (52.4% vs. 29.8%, respectively; OR = 2.59; 95% CI (1.13, 5.94); *p* < 0.05); similarly, there was an 1.8-fold increase in the odds of positive sIgE-mediated food hypersensitivity to nuts in subjects with high physical performance aged > 45 years compared to subjects with low physical performance (50.0% vs. 35.4%, respectively; OR = 1.83; 95% CI (1.10, 3.82); *p* < 0.05). There was a 2.3-fold increase in the odds of positive sIgE-mediated food hypersensitivity to nuts in subjects with high physical performance presenting overweight or a higher BMI compared to subjects with low physical performance (50.0% vs. 31.3%, respectively; OR = 2.27; 95% CI (1.02, 5.31); *p* < 0.05). In contrast, among those subjects with high physical performance whether women and adults aged ≤45 y appeared to be more prone to develop IgG_4_-mediated ARFS to legumes compared to their low physical performance counterparts (women, 76.6% vs. 62.6%, respectively; OR = 1.95; 95% CI (1.03, 3.99); *p* < 0.05); (adults ≤ 45 y, 71.2% vs. 53.0%, respectively; OR = 2.19; 95% CI (1.04, 4.58); *p* < 0.05).

No significant associations were observed for the calculated OR within the lactose intolerance and fructose malabsorption categories. However, the analysis of age groups regarding lactose intolerance and fructose malabsorption revealed that the younger age group with high physical performance was linked to a 2.23-fold rise in the odds of testing negative for both lactose intolerance and fructose malabsorption (59.4% vs. 36.9%, respectively; OR = 2.23; 95% CI (1.04, 5.56); *p* < 0.05).

## 4. Discussion

This study utilized a well-defined group of Spanish adults self-reporting symptoms related to ARFSs to determine potential factors analyzing novel relationships between physical performance and ARFSs. ARFS prevalence was determined, associations between physical performance variables and food allergen-specific AbR were evaluated, and ORs’ associations of high physical performance with the likelihood of presenting an ARFS were explored.

It remains unclear at this point whether sIgG_4_ AbR is associated with disease improvement, involved directly in the allergic response, or serves as an indicator of disease severity and food hypersensitivity. Nevertheless, the mechanisms of sIgG_4_ AbR in the food allergy cascade have not been analyzed in present study. Food allergen-specific sIgG_4_ AbR was classified as type 2 food hypersensitivity associated with ARFS symptoms based on descriptive studies [4,31]. Among the studied food groups, the prevalence of type 2 food hypersensitivity was significantly higher than type 1 food hypersensitivity. Participants with type 1 food hypersensitivity had a higher prevalence of positive sIgE AbR against legumes and seeds, while those with type 2 food hypersensitivity showed a higher prevalence of positive sIgG_4_ AbR against legumes and nuts.

In a previous study conducted in Sweden, it was observed that sensitization to nuts is highly prevalent in adults [32]. In this Swedish cohort, it was found that the prevalence of sIgE AbR against nuts was 21.2% in 24-year-old adults. However, the present research found that adults aged 45 or younger had a prevalence of 38.5% for sIgE AbR against nuts. It was also stated that tree nut type 1 food hypersensitivity is linked to the development of early, long-lasting, and severe allergic conditions in adulthood. Overall, a European systematic review and meta-analysis revealed that food hypersensitivities to certain food groups, previously considered unimportant in adulthood, are now acknowledged as important food hypersensitivities, particularly type 1 food hypersensitivity against fruits, vegetables, seeds, legumes, grains, and cereals [33].

Associations were identified among various physical fitness tests, age groups, sociodemographic descriptions, general sample characteristics, and ARFS biomarker values (type 1 food hypersensitivity, type 2 food hypersensitivity, lactose intolerance, and fructose malabsorption). Generally, cardiorespiratory fitness is measured together with asthma allergic incidence, but data regarding their direct relationship to food hypersensitivity are still lacking [34]. The direct and novel relationship between physical fitness tests and the prevalence of certain positive food hypersensitivity reactions is still unexplored. However, the role that any food hypersensitivity may have in physical performance is clearly different depending on the type of food hypersensitivity and the food group involved in the disease (Figure S1). A study of allergy in English runners showed that the prevalence of allergy (response to environmental allergens) in recreational marathon runners was higher than that in the general population [35]. Considering the “dual allergen exposure hypothesis”, changes in environmental conditions during sports may also contribute to ARFS disease progression, and the discipline and environment around the exercise development might also be of great influence [13].

The food allergen-specific biomarkers of type 1 and type 2 food hypersensitivities, sIgE AbR and sIgG_4_ AbR, were examined in 12 specific food groups and associated with physical performance variables, and they showed significant differences across age groups and the fitness tests which were measured as physical performance variables. In comparison to the older age group, the younger age group showed positivity for fewer food groups when evaluating each physical performance variable against the 12 analyzed food groups. In the older age group, cardiorespiratory fitness revealed significant positive associations in half of the studied food groups when compared to the younger age group. Cardiorespiratory fitness is currently acknowledged as a key factor in studies involving health assessments [36]. Cardiorespiratory fitness has also been recognized as an indicator of inflammation, whether it is acute (infectious illness) or chronic (long-term from other conditions like atherosclerotic heart disease), both of which can affect physical abilities [37]. Previous studies have also found direct connections between osteoarthritis, allergies, and metrics of physical health [38]. Previous research has shown associations between food allergy and osteoarthritis, particularly because patients with milk, meat, and vegetable food allergies often follow restrictive diets low in calcium, magnesium, and other key nutrients which can impact their musculoskeletal and bone health, worsening their physical conditions in the long term [39].

In both children and adults, lactose intolerance is frequently associated with gastrointestinal discomfort. Studies in the adult population were focused on the impact of lactose intolerance on athletes and physically active individuals. Lactose appears to have a significant role in muscle recovery and function as a vehicle for the delivery of glucose and galactose [40]. In the current study, adults with positive lactose intolerance showed significantly reduced handgrip strength values. These results are aligned with previous findings about the strong relationship between handgrip strength and gastrointestinal symptoms, enzymatic food intolerance, such as lactose intolerance, and other chronic diseases [41].

Features associated with food hypersensitivity include assessments such as a clinical history evaluation and an analysis of the type of allergen exposure. In developed countries, physically active adults with limited allergen exposure since childhood may be greatly influenced by the transition from a protected environment to engaging in physical exercise in adulthood when it comes to food hypersensitivity. Some food allergen extracts in certain age groups have a greater impact than others, such as nuts, with a higher prevalence in European adults than children, or legumes, with significant prevalence in Spanish women older than 14 years of age [42].

### 4.1. Strengths and Limitations

#### 4.1.1. Strengths

Significant disparities in levels of physical fitness and exercise have been documented among adults based on race and income. The present paper involves a clearly defined sample of Caucasian Spanish adults, with most of them being employed (>80%) and having received a university education or higher (>70%). The studied sample showed satisfactory homogeneity in terms of demographic characteristics. The methodology of this study adhered to well-known standardized methods for accurately measuring body composition and physical performance variables. The ALASKA study protocol was specifically developed for this study on ARFS and for a physical performance analysis. The staff members of the ImFINE Research group who carried out the ALASKA study evaluations were specialized in physical fitness tests (sports physicians and physical activity and sport science practitioners).

#### 4.1.2. Limitations

The ARFS biomarkers analyzed in this study were focused on type 1 food hypersensitivity, type 2 food hypersensitivity, lactose intolerance, and fructose malabsorption. There might be other interesting biomarkers associated with ARFS. Thus, when analyzing other types of ARFSs, there could be other recommended relevant variables of interest, such as celiac disease. Some other ARFS-related measurements might be the characterization of the microbiota, genetic testing (HLA DQ-2 and DQ-8), and serum biomarkers such as type 2 transglutaminase, deamidated gliadin peptide, gliadins, and microbial transglutaminase.

The findings of this research were based on a group of adults who self-reported PSIMP-ARFSQ-10 scores of 6 or higher. However, further studies are needed to conclude the association between high physical performance and the odds of experiencing an ARFS in the general adult population.

Participants in the ALASKA study mostly met the World Health Organization’s current physical activity recommendations. It would be relevant to perform the same analysis in a population who self-report not meeting these recommendations.

### 4.2. Future Research

Dietary patterns and the type of exercise or sport are important aspects to consider for future research studies regarding ARFSs. Further investigation is needed to determine the potential influence of focused ARFS interventions on the connections between physical performance and overall sample characteristics.

## 5. Conclusions

The most common ARFS in the overall sample of the studied Spanish adults was legumes (50% and 64%, respectively, for type 1 and type 2 food hypersensitivities). Adults aged 45 y or younger had the most prevalent sIgE-mediated or type 1 food hypersensitivities to legumes (53%). Analogously, adults older than 45 y of age presented the most prevalent sIgG_4_-mediated or type 2 food hypersensitivities to legumes (68%). The total positive sIgG_4_ AbR was associated with higher values of physical performance and was significantly related with higher values of body balance compared to negative sIgG_4_ AbR. Non-immune-mediated reactions or food intolerances were linked to lower values of physical performance; specifically, lactose intolerance was inversely associated with maximal handgrip strength. The probability of developing total positive sIgE immune-mediated reactions or type 1 food hypersensitivity is higher in subjects presenting better physical performance. Age, sex, and specific involved food groups (nuts, seeds, legumes, cereals, and fruits) might be factors of crucial influence when evaluating the odds of developing an ARFS in adults with high physical performance. Further research is needed to clarify the relationship between physical performance-related variables and ARFSs.

## Figures and Tables

**Table 1:** A summary of the variables and research instruments of the cross-sectional analysis of the ALASKA study.

	Variables/Assessments		Questionnaires/Instruments	References
Sample characteristics	Participant consent		Informed consent form	[3]
	Eligibility screening		Inclusion and exclusion criteria form	[3]
	Demographics and clinical information		Demographics and clinical characteristics form	[3]
	Diseases, signs, and symptoms		PSIMP-ARFSQ-10	[14]
	Height (cm)		Stadiometer	(Seca GmbH & Co. KG., model 213 1721009, Hamburg, Germany)
	Waist/hip ratio (ISAK criteria)		Anthropometric tape	(Cescorf Ltd., 2 m Innovare measuring tape, Porto Alegre City, Brazil)
	Weight (kg) and BIA with segmental multifrequency		BIA bascule	(Tanita, model MC-780MA-N, Tokyo, Japan)
	Body mass index (kg/m^2^, mass in kilograms divided by height in meters squared)		Height and weight instruments	[15,16]
	Physical activity		IPAQ-LF	[17,18]
			RAPA	[19]
Clinical analysis	Recommended practices and requirements for participants before blood collection		Blood Sample Questionnaire	[3]
	T1FH, food allergen-specific sIgE AbR		In-line immunoassay analyzer	(Aesku.Diagnostics GmbH, Helia^®^ Helmed, Wendelsheim, Germany)
	T2FH, food allergen-specific sIgG_4_ AbR			
	Lactose intolerance		Lactose hydrogen and methane breath test kit	(Cerascreen GmbH, Cerascreen^®^ Lactose kit, Schwerin, Germany)
	Fructose malabsorption		Fructose hydrogen and methane breath test kit	(Cerascreen GmbH, Cerascreen^®^ Fructose kit, Schwerin, Germany)
Physical performance	Resting heart rate		Tensiometer	(Omron Healthcare Co. Ltd., model M3 Comfort HEM-7155-E(C), Kyoto, Japan)
	Handgrip (hand/upper extremity strength)		Dynamometer	(Takei, model T.K.K. 5401 Grip-D, Tokyo, Japan)
	Power (leg/lower extremity strength)		Force platform	(Tanita, model BM-220 7341(0), Tokyo, Japan)
	Sit-to-stand speed			
	Body balance			
	Cardiorespiratory fitness	Estimated VO_2_max	Åstrand-Ryhming Step Test	[20]
		Pulse rate	Pulse oximeter	(GE Healthcare, Ohmeda Tuffsat, Boston, MA, USA)
		Pace/cycles	Metronome	(KORG Inc., model MA-1, Tokyo, Japan)

BIA, bioelectrical impedance analysis; IPAQ-LF, International Physical Activity Questionnaire Long Form; ISAK, International Society for the Advancement of Kinanthropometry; PSIMP-ARFSQ-10, Pathologies and Symptomatology Questionnaire associated with Adverse Reactions to Foodstuffs; RAPA, Rapid Assessment of Physical Activity Questionnaire; T1FH, type 1 food hypersensitivity; T2FH, type 2 food hypersensitivity; VO_2_max, maximal oxygen consumption.

**Table 2:** Sample characteristics of the ALASKA study compared by sex.

	Mean ± SD or *n* (%)				
	Total (*n* = 254)	Men (*n* = 99)	Women (*n* = 155)	Min-Max	*p*-Values_sex_
Education level					
Highschool	27 (10.6)	10 (10.1)	17 (11.0)	--	*NS* 0.275
Intermediate-level professional training	11 (4.3)	2 (2.0)	9 (5.8)	--	
Higher-level professional training	36 (14.2)	16 (16.2)	20 (12.9)	--	
University education (college graduate, engineer, high degree)	105 (41.3)	41 (41.4)	64 (41.3)	--	
Master’s degree	60 (23.7)	23 (23.2)	37 (23.9)	--	
PhD/doctorate’s degree	15 (5.9)	7 (7.1)	8 (5.2)	--	
Job situation					
Employed or freelance	209 (82.3)	86 (86.9)	123 (79.4)	--	*NS* 0.140
Learning period paid student	5 (2.0)	1 (1.0)	4 (2.6)	--	
Learning period non-paid student	12 (4.7)	3 (3.0)	9 (5.8)	--	
Unemployed	7 (2.8)	2 (2.0)	5 (3.2)	--	
Retired or on early retirement	9 (3.5)	4 (4.0)	5 (3.2)	--	
Permanent disability individual	1 (0.4)	1 (0.4)	1 (0.6)	--	
Homemaker or houseworker (housewife/husband)	5 (2.0)	0 (0.0)	5 (3.2)	--	
Other	6 (2.4)	3 (3.0)	3 (1.9)	--	
Living situation					
Number of people living with (NPLW)	2.05 ± 1.27	2.03 ± 1.31	2.06 ± 1.24	0.00–6.00	*NS* 0.433
≤2 NPLW	162 (63.8)	65 (65.7)	97 (62.6)	--	*NS* 0.620
>2 NPLW	92 (36.2)	34 (34.3)	58 (37.4)	--	
Anthropometry					
Body mass index (kg/m^2^)	24.40 ± 4.54	25.17 ± 3.80	23.90 ± 4.89	17.10–41.00	<0.05
Height (cm)	168.70 ± 9.08	176.67 ± 6.68	163.60 ± 6.32	149.00–196.00	<0.001
Weight (kg)	69.59 ± 14.64	78.65 ± 13.18	63.80 ± 12.46	41.80–124.80	<0.001
Waist circumference (cm)	79.62 ± 12.74	87.19 ± 11.04	74.78 ± 11.35	38.00–126.80	<0.001
Hip circumference (cm)	97.02 ± 12.06	97.95 ± 8.25	96.42 ± 13.96	63.00–192.00	*NS* 0.163
Waist-to-hip ratio	0.82 ± 0.10	0.89 ± 0.07	0.78 ± 0.09	0.32–1.36	<0.001
Body mass index					
<18.5 underweight (%)	5 (2.0)	1 (1.0)	4 (2.6)	--	*NS* 0.220
18.5–24.9 normal weight (%)	163 (64.2)	57 (57.6)	106 (68.4)	--	
25.0–29.9 overweight (%)	63 (24.8)	34 (34.3)	29 (18.7)	--	
30.0–34.9 class I obesity (%)	9 (3.5)	3 (3.0)	6 (3.9)	--	
35.0–39.9 class II obesity (%)	14 (5.5)	4 (4.0)	10 (6.5)	--	
Body composition					
Fat mass (%)	25.08 ± 8.63	20.48 ± 6.62	28.02 ± 8.50	5.60–51.60	<0.001
Fat mass (kg)	17.93 ± 9.07	16.74 ± 8.07	18.68 ± 9.61	3.90–51.00	<0.05
Fat free mass (kg)	51.66 ± 10.12	61.91 ± 6.97	45.12 ± 5.18	31.20–79.30	<0.001
Muscle mass (kg)	49.06 ± 9.64	58.82 ± 6.64	42.82 ± 4.92	29.60–75.40	<0.001
Bone mass (kg)	2.60 ± 0.48	3.09 ± 0.33	2.29 ± 0.26	1.60–3.90	<0.001
Body water (%)	51.89 ± 5.95	56.27 ± 4.13	49.10 ± 5.21	35.90–66.60	<0.001
Body water (kg)	35.85 ± 7.38	43.80 ± 4.56	30.77 ± 3.10	24.30–56.90	<0.001
Visceral fat rating (VFR ^1^)	5.96 ± 4.18	8.27 ± 4.43	4.48 ± 3.25	1.00–23.00	<0.001

^1^ VFR, score ranges from 1 to 59 using the Tanita, model MC-780MA-N, Tokyo, Japan. *NS*, statistically non-significant.

**Table 3:** Symptomatology, physical activity, and physical performance compared by sex and age.

	Mean ±SD or *n* (%)													
	Total (*n* = 254)	Men (*n* = 99)	Women (*n* = 155)	Min-Max	*p*-Values_sex_	≤45 y (*n* = 135)			>45 y (*n* = 119)			*p*- Values_age_	*p*- Values_age-men_	*p*- Values_age-women_
						Total ≤ 45 y	Men (*n* = 43)	Women (*n* = 92)	Total > 45 y	Men (*n* = 56)	Women (*n* = 63)			
Age (years)	43.71 ± 12.61	46.23 ± 13.04	42.10 ± 12.10	18.00–77.00	<0.05	33.77 ± 7.55	33.91 ± 7.94	33.71 ± 7.40	54.99 ± 5.90	55.70 ± 6.68	54.37 ± 5.08	<0.001	<0.001	<0.001
Symptomatology ^1^														
Digestive system	4.87 ± 2.77	4.77 ± 2.71	4.94 ± 2.81	0.00–15.00	*NS* 0.319	5.29 ± 2.56	4.98 ± 2.56	5.43 ± 2.56	4.40 ± 2.93	4.61 ± 2.83	4.21 ± 3.02	<0.01	*NS* 0.252	<0.05
Skin and subcutaneous tissue	2.92 ± 2.65	2.64 ± 2.54	3.10 ± 2.71	0.00–14.00	*NS* 0.089	2.84 ± 2.48	2.70 ± 2.19	2.91 ± 2.62	3.00 ± 2.84	2.59 ± 2.79	3.37 ± 2.85	*NS* 0.321	*NS* 0.417	*NS* 0.155
Nervous system	3.04 ± 2.75	2.07 ± 2.34	3.66 ± 2.83	0.00–16.00	<0.001	3.36 ± 2.76	2.44 ± 2.35	3.79 ± 2.85	2.67 ± 2.71	1.79 ± 2.31	3.46 ± 2.82	<0.05	*NS* 0.084	*NS* 0.236
Respiratory system	1.56 ± 1.86	1.70 ± 1.84	1.47 ± 1.87	0.00–11.00	*NS* 0.173	1.47 ± 1.69	1.77 ± 1.48	1.33 ± 1.77	1.66 ± 2.04	1.64 ± 2.08	1.68 ± 2.01	*NS* 0.200	*NS* 0.370	*NS* 0.123
Other pathologies/ diseases	2.49 ± 2.34	2.14 ± 2.13	2.72 ± 2.45	0.00–10.00	<0.05	1.81 ± 2.00	1.42 ± 1.79	1.99 ± 2.07	3.27 ± 2.47	2.70 ± 2.22	3.78 ± 2.58	<0.001	=0.001	<0.001
Total PSIMP-ARFSQ-10 score	14.88 ± 7.97	13.31 ± 7.33	15.88 ± 8.21	6.00–44.00	<0.05	14.77 ± 7.46	13.30 ± 6.67	15.46 ± 7.74	15.00 ± 8.53	13.32 ± 7.86	16.49 ± 8.88	*NS* 0.410	*NS* 0.495	*NS* 0.221
Physical activity														
Meet WHO recommendations	219 (86.2)	85 (85.9)	134 (86.5)	--	*NS* 0.447	119 (88.1)	39 (90.7)	80 (87.0)	100 (84.0)	46 (82.1)	54 (85.7)	*NS* 0.172	*NS* 0.115	*NS* 0.413
Do not meet WHO recommendations	35 (13.8)	14 (14.1)	21 (13.5)	--		16 (11.9)	4 (9.3)	12 (13.0)	19 (16.0)	10 (17.9)	9 (14.3)			
Physical performance														
Total maximal HGS (kg)	34.46 ± 9.91	44.41 ± 6.30	28.11 ± 5.66	17.30–66.00	<0.001	34.18 ± 9.96	45.36 ± 6.39	28.95 ± 6.36	34.78 ± 9.90	43.68 ± 6.18	26.88 ± 4.20	*NS* 0.314	*NS* 0.095	<0.05
Leg power (f/w)	1.55 ± 0.24	1.61 ± 0.26	1.51 ± 0.23	1.10–2.39	<0.001	1.65 ± 0.25	1.78 ± 0.22	1.58 ± 0.23	1.44 ± 1.19	1.47 ± 0.20	1.41 ± 0.17	<0.001	<0.001	<0.001
Sit-to-stand speed (RFD/w)	12.61 ± 3.75	12.79 ± 4.10	12.49 ± 3.51	0.01–23.60	*NS* 0.271	13.85 ± 3.58	15.27 ± 3.23	13.18 ± 3.55	11.20 ± 3.43	10.88 ± 3.66	11.49 ± 3.23	<0.001	<0.001	=0.001
Body balance (BP T-score)	50.12 ± 9.42	49.43 ± 10.46	50.55 ± 8.69	1.00–63.00	*NS* 0.178	50.43 ± 8.61	50.33 ± 8.33	50.48 ± 8.77	49.76 ± 10.28	48.75 ± 11.86	50.67 ± 8.64	*NS* 0.575	*NS* 0.230	*NS* 0.448
Estimated VO_2_max (ml/kg·min)	39.25 ± 9.92	42.79 ± 10.06	36.98 ± 9.17	21.45–68.96	<0.001	43.28 ± 9.39	48.80 ± 8.98	40.70 ± 8.44	34.67 ± 8.45	38.18 ± 8.29	31.55 ± 7.34	<0.001	<0.001	<0.001
Low CRF (%)	46 (18.1)	15 (15.2)	31 (20.0)	--	*NS* 0.139	15 (11.1)	2 (4.7)	13 (14.1)	31 (26.1)	13 (23.2)	18 (28.6)	<0.05	<0.05	*NS* 0.092
Normal CRF (%)	68 (26.8)	26 (26.3)	42 (27.1)	--		39 (28.9)	11 (25.6)	28 (30.4)	29 (24.4)	15 (26.8)	14 (22.2)			
Good CRF (%)	115 (45.3)	47 (47.5)	68 (43.9)	--		66 (48.9)	23 (53.5)	43 (46.7)	49 (41.2)	24 (42.9)	25 (39.7)			
Excellent CRF (%)	25 (9.8)	11 (11.1)	14 (9.0)	--		15 (11.1)	7 (16.3)	8 (8.7)	10 (8.4)	4 (7.1)	6 (9.5)			

^1^ Number of pathologies/diseases, signs, and symptoms related to ARFS [14]. BP, body power; f, force; CRF, cardiorespiratory fitness; HGS, handgrip strength; *NS*, statistically non-significant; PSIMP-ARFSQ-10, Pathologies and Symptomatology Questionnaire associated with Adverse Re-actions to Foodstuffs; RFD, Rate of Force Development in N/s; T-score, bone health indicator; VO_2_max, maximal oxygen consumption; w, weight; WHO, World Health Organization.

## Data Availability

The original contributions presented in this study are included in this article and supplementary material; further inquiries can be directed to the corresponding author, Lisset Pantoja-Arévalo, l.pantoja@upm.es (L.P.-A.), and to the principal investigator (PI) of this study, Marcela González-Gross, marcela.gonzalez.gross@upm.es (M.G.-G.).

## Supplementary Materials

The following supporting information can be downloaded at https://www.mdpi.com/article/10.3390/nu16244384/s1, Table S1: Food-specific immunoglobulin E antibody reactions compared based on sex and age; Table S2: Food-specific immunoglobulin G_4_ antibody reactions compared based on sex and age; Table S3: Summary of adverse reactions to food types by sex and age; Figure S1: Physical performance variables and adverse reactions to foodstuff against 12 food groups of total studied sample of Spanish adults; Figure S2: Physical performance variables and adverse reactions to foodstuff against 12 food groups of Spanish adults older than 45 y of age.

## References

[1] Tanno L.K., Chalmers R., Jacob R., Kostanjsek N., Bierrenbach A.L., Martin B., Molinari N., Annesi-Maesano I., Papadopoulos N.G., Sanchez-Borges M. (2020). Global implementation of the World Health Organization’s International Classification of Diseases (ICD)-11: The allergic and hypersensitivity conditions model. Allergy.

[2] Spolidoro G.C.I., Amera Y.T., Ali M.M., Nyassi S., Lisik D., Ioannidou A., Rovner G., Khaleva E., Venter C., van Ree R. (2023). Frequency of food allergy in Europe: An updated systematic review and meta-analysis. Allergy.

[3] Pantoja-Arévalo L., Gesteiro E., Pérez-Ruiz M., López-Seoane J., Wusterhausen P., Matthias T., Urrialde R., González-Gross M. (2024). The multifactorial approach and the food allergen-specific substitutive diet as a tool to manage and ameliorate adverse reactions to foodstuffs in adulthood: Study protocol for a randomized controlled trial—The ALASKA Study. Trials.

[4] Pantoja-Arévalo L., Gesteiro E., Matthias T., Urrialde R., González-Gross M. (2023). Association between food-specific immunoglobulin G4 antibodies in adults with self-reported signs and symptoms attributed to adverse reactions to foodstuffs. Biomedicines.

[5] Santos A.F., Riggioni C., Agache I., Akdis C.A., Akdis M., Alvarez-Perea A., Alvaro-Lozano M., Ballmer-Weber B., Barni S., Beyer K. (2023). EAACI guidelines on the diagnosis of IgE-mediated food allergy. Allergy.

[6] Misselwitz B., Butter M., Verbeke K., Fox M.R. (2019). Update on lactose malabsorption and intolerance: Pathogenesis, diagnosis and clinical management. Gut.

[7] Aldred S., Love J.A., Tonks L.A., Stephens E., Jones D.S., Blannin A.K. (2010). The effect of steady state exercise on circulating human IgE and IgG in young healthy volunteers with known allergy. J. Sci. Med. Sport..

[8] Antunes L., Meirelles M.D.L., Moreira D., Menezes Z., Alves D., Vieira N., Batista G., Versiani A., Da-Gloria D., Carmona D. (2011). Experimental food allergy leads to adipose tissue inflammation, systemic metabolic alterations and weight loss in mice. Cell Immunol..

[9] Sicherer S.H., Sampson H.A. (2010). Food allergy. J. Allergy Clin. Immunol..

[10] Nowak-Wegrzyn A., Szajewska H., Lack G. (2017). Food allergy and the gut. Nat. Rev. Gastroenterol. Hepatol..

[11] Tham E.H., Leung D.Y.M. (2018). How Different Parts of the World Provide New Insights Into Food Allergy. Allergy Asthma Immunol. Res..

[12] Lee E.C.K., Trogen B., Brady K., Ford L.S., Wang J. (2024). The Natural History and Risk Factors for the Development of Food Allergies in Children and Adults. Curr. Allergy Asthma Rep..

[13] Kulis M.D., Smeekens J.M., Immormino R.M., Moran T.P. (2021). The airway as a route of sensitization to peanut: An update to the dual allergen exposure hypothesis. J. Allergy Clin. Immunol..

[14] Pantoja-Arévalo L., Gesteiro E., Calonge-Pascual S., Pérez-Ruiz M., Urrialde R., González-Gross M. (2023). Design and validity of the Spanish version of two questionnaires related to adverse reactions to foodstuffs. Nutr. Hosp..

[15] Kolimechkov S., Petrov L. (2020). The Body Mass Index: A systematic review. J. Exerc. Physiol..

[16] Weir C.B., Jan A. (2023). BMI Classification Percentile and Cut off Points.

[17] Craig C.L., Marshall A.L., Sjostrom M., Bauman A.E., Booth M.L., Ainsworth B.E., Pratt M., Ekelund U., Yngve A., Sallis J.F. (2003). International physical activity questionnaire: 12-country reliability and validity. Med. Sci. Sports Exerc..

[18] Roman-Viñas B., Serra-Majem L., Hagströmer M., Ribas-Barba L., Sjöström M., Segura-Cardona R. (2010). International physical activity questionnaire: Reliability and validity in a Spanish population. Eur. J. Sport. Sci..

[19] Topolski T.D., LoGerfo J., Patrick D.L., Williams B., Walwick J., Patrick M.B. (2006). The Rapid Assessment of Physical Activity (RAPA) among older adults. Prev. Chronic Dis..

[20] Maud P.J., Foster C. (2006). Physiological Assessment of Human Fitness.

[21] Sun B.Q., Zheng P.Y., Zhang X.W., Huang H.M., Chen D.H., Zeng G.Q. (2014). Prevalence of allergen sensitization among patients with allergic diseases in Guangzhou, Southern China: A four-year observational study. Multidiscip. Respir. Med..

[22] Gupta R.S., Warren C.M., Smith B.M., Jiang J., Blumenstock J.A., Davis M.M., Schleimer R.P., Nadeau K.C. (2019). Prevalence and Severity of Food Allergies Among US Adults. JAMA Netw. Open.

[23] Jarvis D., Luczynska C., Chinn S., Potts J., Sunyer J., Janson C., Svanes C., Kunzli N., Leynaert B., Heinrich J. (2005). Change in prevalence of IgE sensitization and mean total IgE with age and cohort. J. Allergy Clin. Immunol..

[24] World-Health-Organization (2020). WHO Guidelines on Physical Activity and Sedentary Behaviour.

[25] Myers J., Prakash M., Froelicher V., Do D., Partington S., Atwood J.E. (2002). Exercise capacity and mortality among men referred for exercise testing. N. Engl. J. Med..

[26] Hofstee W.K.B., Berge J.M.F.T., Hendriks A.A.J. (1998). How to score questionnaires. Personal. Individ. Differ..

[27] Goodwin G., Ryu S. (2023). Understanding The Odds: Statistics in Public Health. Front. Young Minds.

[28] Szumilas M. (2010). Explaining odds ratios. J. Can. Acad. Child. Adolesc. Psychiatry.

[29] World Medical Association (2013). World Medical Association Declaration of Helsinki: Ethical principles for medical research involving human subjects. JAMA.

[30] European Parliament (2016). Regulation (EU) 2016/679 of the European Parliament and of the Council of 27 April 2016 on the protection of natural persons with regard to the processing of personal data and on the free movement of such data, repealing Directive 95/46/EC. OJEU.

[31] Zar S., Mincher L., Benson M.J., Kumar D. (2005). Food-specific IgG4 antibody-guided exclusion diet improves symptoms and rectal compliance in irritable bowel syndrome. Scand. J. Gastroenterol..

[32] Bager J., Tedner S.G., Andersson N., Ballardini N., Borres M.P., Konradsen J.R., Nilsson C., Westman M., Kull I., Bergstrom A. (2021). Prevalence and early-life risk factors for tree nut sensitization and allergy in young adults. Clin. Exp. Allergy.

[33] Spolidoro G.C.I., Nyassi S., Lisik D., Ioannidou A., Ali M.M., Amera Y.T., Rovner G., Khaleva E., Venter C., van Ree R. (2024). Food allergy outside the eight big foods in Europe: A systematic review and meta-analysis. Clin. Transl. Allergy.

[34] Ortega F.B., Lee D.C., Sui X., Ruiz J.R., Cheng Y.J., Church T.J., Miller C.C., Blair S.N. (2010). Cardiorespiratory fitness, adiposity, and incident asthma in adults. J. Allergy Clin. Immunol..

[35] Robson-Ansley P., Howatson G., Tallent J., Mitcheson K., Walshe I., Toms C., Du Toit G., Smith M., Ansley L. (2012). Prevalence of allergy and upper respiratory tract symptoms in runners of the London marathon. Med. Sci. Sports Exerc..

[36] Kaminsky L.A., Arena R., Ellingsen O., Harber M.P., Myers J., Ozemek C., Ross R. (2019). Cardiorespiratory fitness and cardiovascular disease—The past, present, and future. Prog. Cardiovasc. Dis..

[37] Arsenault B.J., Cartier A., Cote M., Lemieux I., Tremblay A., Bouchard C., Perusse L., Despres J.P. (2009). Body composition, cardiorespiratory fitness, and low-grade inflammation in middle-aged men and women. Am. J. Cardiol..

[38] Malsagova K.A., Kopylov A.T., Stepanov A.A., Enikeev D.V., Potoldykova N.V., Balakin E.I., Pustovoyt V.I., Kaysheva A.L. (2023). Molecular profiling of athletes performing high-intensity exercises in extreme environments. Sports.

[39] Skypala I.J., McKenzie R. (2019). Nutritional issues in food allergy. Clin. Rev. Allergy Immunol..

[40] Odell O.J., Wallis G.A. (2021). The application of lactose in sports nutrition. Int. Dairy. J..

[41] Cheung C.L., Nguyen U.S., Au E., Tan K.C., Kung A.W. (2013). Association of handgrip strength with chronic diseases and multimorbidity: A cross-sectional study. Age.

[42] Somoza M.L., Blanca-López N., Alzate D.P., Garcimartin M.I., Ruano F.J., Antón-Laiseca A., Canto G. (2015). Allergy to Legumes in Adults: Descriptive Features. J. Allergy Clin. Immunol..

