# Supplementary material for: Is There a Relationship Between Physical Performance Factors and Adverse Reactions to Foodstuffs? The ALASKA Study
Source: Nutrients. 2024 Dec 20;16(24):4384. doi: 10.3390/nu16244384 (PMC11676144; doi:10.3390/nu16244384)
Supplement: Supplementary file 1 [file nutrients-16-04384-s001.zip › nutrients-3341879-supplementary.pdf]

SUPPLEMENTARY MATERIALS

SUPPLEMENTARY TABLES AND FIGURES

Table S1. Food-specific Immunoglobulin E antibody reactions compared by sex and age

	M±SD or *n* (%)													
	**Total (*n*=254)**	**Men (*n*=99)**	**Women (*n*=155)**	**Min-Max**	***p*-values$_{sex}$**	**≤45y (*n*=135)**			**>45y (*n*=119)**			***p*-values$_{age}$**	***p*-values$_{age-men}$**	***p*-values$_{age-women}$**
						**Total ≤45y**	**Men (*n*=43)**	**Women (*n*=92)**	**Total >45y**	**Men (*n*=56)**	**Women (*n*=63)**			
Age (years)	43.71±12.61	46.23±13.04	42.10±12.10	18.00-77.00	SIG <0.05	33.77±7.55	33.91±7.94	33.71±7.40	54.99±5.90	55.70±6.68	54.37±5.08	SIG <0.001	SIG <0.001	SIG <0.001
**sIgE AbR**														
Total sIgE$_{CALCULATED}$ [a] AbR (kUA/l)	4.22±4.87	4.11±4.41	4.28±5.15	0.12-20.65	NS 0.394	4.56±4.87	4.84±5.21	4.43±5.78	3.83±3.87	3.56±3.64	4.08±4.09	NS 0.118	NS 0.076	NS 0.339
+ (%) [b]	100 (39.4)	41 (41.4)	59 (38.1)	--	NS 0.298	52 (38.5)	20 (46.5)	32 (34.8)	48 (40.3)	21 (37.5)	48 (40.3)	NS 0.384	NS 0.186	NS 0.156
Legume IgE AbR (kUA/l)	11.51±16.66	11.98±17.17	11.20±16.38	0.16-54.98	NS 0.359	12.87±17.25	15.23±18.84	11.77±16.44	9.96±15.90	9.49±15.49	10.37±16.38	NS 0.082	NS 0.050	NS 0.302
+ (%)	126 (49.6)	49 (49.5)	77 (49.7)	--	NS 0.489	71 (52.6)	24 (55.8)	47 (51.1)	55 (46.2)	25 (44.6)	55 (46.2)	NS 0.156	NS 0.138	NS 0.337
Seeds IgE AbR (kUA/l)	7.28±11.75	7.57±11.97	7.09±11.64	0.10-81.39	NS 0.374	8.40±12.73	11.20±15.39	7.10±11.13	6.00±10.43	4.79±7.51	7.07±12.43	NS 0.052	SIG <0.01	NS 0.495
+ (%)	110 (43.3)	45 (45.5)	65 (41.9)	--	NS 0.291	61 (45.2)	24 (55.8)	37 (40.2)	49 (41.2)	21 (37.5)	49 (41.2)	NS 0.261	SIG <0.05	NS 0.302
Cereal IgE AbR (kUA/l)	5.84±11.44	6.46±11.11	5.44±11.67	0.11-86.76	NS 0.244	7.14±14.10	7.59±13.59	6.93±14.40	4.37±7.13	5.60±8.77	3.27±5.09	SIG <0.05	NS 0.190	SIG <0.05
+ (%)	82 (32.3)	28 (28.3)	54 (34.8)	--	NS 0.139	50 (37.0)	15 (34.9)	35 (38.0)	32 (26.9)	13 (23.2)	32 (26.9)	SIG <0.05	NS 0.103	NS 0.157
Nuts IgE AbR (kUA/l)	5.60±7.57	4.85±6.23	6.08±8.29	0.13-37.11	NS 0.104	5.97±8.36	5.95±7.56	5.98±8.74	5.19±6.57	4.02±4.89	6.23±7.65	NS 0.207	NS 0.064	NS 0.426
+ (%)	102 (40.2)	39 (39.4)	63 (40.6)	--	NS 0.422	52 (38.5)	19 (44.2)	33 (35.9)	50 (42.0)	20 (35.7)	50 (42.0)	NS 0.286	NS 0.199	NS 0.073
Gluten-free cereal IgE AbR (kUA/l)	4.54±11.20	3.70±8.94	5.07±12.42	0.09-100.00	NS 0.172	5.27±13.60	3.74±9.33	5.98±15.19	3.71±7.57	3.67±8.71	3.75±6.47	NS 0.135	NS 0.484	NS 0.137
+ (%)	62 (24.4)	20 (20.2)	42 (27.1)	--	NS 0.107	31 (23.0)	7 (16.3)	24 (26.1)	31 (26.1)	13 (23.2)	31 (26.1)	NS 0.285	NS 0.200	NS 0.367
Fish IgE AbR (kUA/l)	3.57±9.14	2.94±6.62	3.97±10.44	0.12-92.41	NS 0.192	3.54±7.41	3.53±6.66	3.54±7.77	3.60±10.81	2.49±6.61	4.58±13.48	NS 0.479	NS 0.221	NS 0.272
+ (%)	48 (18.9)	18 (18.2)	30 (19.4)	--	NS 0.408	29 (21.5)	11 (25.6)	18 (19.6)	19 (16.0)	7 (12.5)	19 (16.0)	NS 0.132	SIG <0.05	NS 0.468
Meat IgE AbR (kUA/l)	3.68±9.18	3.07±7.26	4.07±10.23	0.07-66.73	NS 0.199	2.75±5.34	2.52±4.40	2.85±5.75	4.75±12.10	3.50±8.88	5.86±14.35	SIG <0.05	NS 0.255	SIG <0.05
+ (%)	50 (19.7)	17 (17.2)	33 (21.3)	--	NS 0.211	35 (25.9)	10 (23.3)	25 (27.2)	15 (12.6)	7 (12.5)	15 (12.6)	SIG <0.01	SIG <0.01	SIG <0.05
Vegetables IgE AbR (kUA/l)	2.23±5.78	1.93±4.46	2.43±6.50	0.10-55.92	NS 0.252	2.22±5.39	1.84±5.25	2.39±5.47	2.25±6.23	2.00±3.79	2.48±7.80	NS 0.481	NS 0.433	NS 0.468
+ (%)	36 (14.2)	13 (13.1)	23 (14.8)	--	NS 0.352	19 (14.1)	4 (9.3)	15 (16.3)	17 (14.3)	9 (16.1)	17 (14.3)	NS 0.481	NS 0.081	NS 0.269
Shellfish/ mollusks IgE AbR (kUA/l)	2.22±4.62	2.14±4.25	2.28±4.85	0.09-47.90	NS 0.409	2.75±5.73	2.72±5.90	2.76±5.69	1.63±2.79	1.70±2.30	1.57±3.19	SIG <0.05	NS 0.119	NS 0.066
+ (%)	43 (16.9)	11 (11.1)	32 (20.6)	--	SIG <0.05	24 (17.8)	4 (9.3)	20 (21.7)	19 (16.0)	7 (12.5)	19 (16.0)	NS 0.351	NS 0.164	NS 0.343
Milk IgE AbR (kUA/l)	1.74±6.17	1.90±6.56	1.64±5.93	0.00-46.60	NS 0.373	1.57±5.46	1.04±2.44	1.81±6.40	1.94±6.91	2.56±8.43	1.39±5.22	NS 0.316	NS 0.127	NS 0.331
+ (%)	22 (8.7)	8 (8.1)	14 (9.0)	--	NS 0.397	13 (9.6)	4 (9.3)	9 (9.8)	9 (7.6)	4 (7.1)	9 (7.6)	NS 0.280	NS 0.310	NS 0.348
Fruits IgE AbR (kUA/l)	1.51±2.97	1.68±3.39	1.40±2.67	0.10-23.94	NS 0.230	1.66±3.02	1.97±3.64	1.52±2.69	1.33±2.91	1.46±3.19	1.22±2.65	NS 0.189	NS 0.233	NS 0.245
+ (%)	31 (12.2)	14 (14.1)	17 (11.0)	--	NS 0.227	20 (14.8)	7 (16.3)	13 (14.1)	11 (9.2)	7 (12.5)	11 (9.2)	NS 0.089	NS 0.350	NS 0.065
Egg IgE AbR (kUA/l)	0.90±2.96	1.14±3.34	0.75±2.70	0.10-29.98	NS 0.148	0.59±1.22	0.80±1.69	0.48±0.91	1.26±4.11	1.41±4.18	1.13±4.08	SIG <0.05	NS 0.187	NS 0.073
+ (%)	13 (5.1)	5 (5.1)	8 (5.2)	--	NS 0.484	9 (6.7)	2 (4.7)	7 (7.6)	4 (3.4)	3 (5.4)	4 (3.4)	NS 0.117	NS 0.299	SIG <0.05

[a] sIgE$_{CALCULATED}$: mean serum IgE AbR. [b] (+) Percentage of positive food-specific serum (sIgE) antibody reactions (≥3.5kUA/l).
AbR: antibody reactions.

Table S2. Food-specific Immunoglobulin G4 antibody reactions compared by sex and age

	M±SD or *n* (%)													
	Total (*n*=254)	Men (*n*=99)	Women (*n*=155)	Min-Max	*p*-values sex	≤45y (*n*=135) Total ≤45y	≤45y Men (*n*=43)	≤45y Women (*n*=92)	>45y (*n*=119) Total >45y	>45y Men (*n*=56)	>45y Women (*n*=63)	*p*-values age	*p*-values age-men	*p*-values age-women
**$sIgG_4$ AbR**														
Total $sIgG_{4CALCULATED}$ [a] AbR (kUA/l)	13.65±13.93	16.24±16.18	12.00±12.04	0.04-77.80	SIG <0.01	14.37±14.12	17.14±15.58	13.07±13.28	12.84±13.71	15.54±16.73	10.43±9.86	NS 0.191	NS 0.314	NS 0.090
+ (%) [b]	185 (72.8)	68 (68.7)	117 (75.5)	--	NS 0.118	105 (77.8)	33 (76.7)	72 (78.3)	80 (67.2)	35 (62.5)	45 (71.4)	SIG <0.05	NS 0.066	NS 0.167
Legume IgG_4 AbR (kUA/l)	17.60±20.46	18.07±24.18	17.30±17.76	0.06-100.00	NS 0.385	16.46±19.38	16.86±22.35	16.27±17.95	18.90±21.63	19.01±25.65	18.81±17.52	NS 0.172	NS 0.332	NS 0.192
+ (%)	162 (63.8)	56 (56.6)	106 (68.4)	--	SIG <0.05	81 (60.0)	24 (55.8)	57 (62.0)	81 (68.1)	32 (57.1)	49 (77.8)	NS 0.092	NS 0.448	SIG <0.05
Seeds IgG_4 AbR (kUA/l)	10.04±16.97	13.54±19.56	7.80±14.72	0.01-83.21	SIG <0.01	9.87±17.46	12.45±18.88	8.66±16.72	10.23±16.47	14.39±20.19	6.54±11.18	NS 0.432	NS 0.314	NS 0.190
+ (%)	92 (36.2)	45 (45.5)	47 (30.3)	--	SIG <0.01	47 (34.8)	20 (46.5)	27 (29.3)	45 (37.8)	25 (44.6)	20 (31.7)	NS 0.311	NS 0.427	NS 0.376
Cereal IgG_4 AbR (kUA/l)	20.83±30.11	25.85±34.37	17.62±26.66	0.00-100.00	SIG <0.05	23.07±29.68	29.38±33.20	20.12±27.59	18.29±30.51	23.14±35.30	13.98±25.02	NS 0.104	NS 0.187	NS 0.080
+ (%)	120 (47.2)	50 (50.5)	70 (45.2)	--	NS 0.204	74 (54.8)	27 (62.8)	47 (51.1)	46 (38.7)	23 (41.1)	23 (36.5)	SIG <0.01	SIG <0.05	SIG <0.05
Nuts IgG_4 AbR (kUA/l)	17.23±20.70	22.77±24.40	13.69±17.10	0.02-85.98	SIG <0.001	17.00±20.19	22.22±23.40	14.56±18.13	17.49±21.34	23.20±25.35	12.43±15.54	NS 0.424	NS 0.422	NS 0.224
+ (%)	156 (61.4)	66 (66.7)	90 (58.1)	--	NS 0.085	85 (63.0)	28 (65.1)	57 (62.0)	71 (59.7)	38 (67.9)	33 (52.4)	NS 0.296	NS 0.389	NS 0.119
Gluten-free cereal IgG_4 AbR (kUA/l)	8.29±16.23	12.23±19.42	5.78±13.28	0.02-100.00	SIG <0.001	8.38±16.91	14.42±22.15	5.56±13.02	8.18±15.48	10.54±17.04	6.09±13.75	NS 0.461	NS 0.164	NS 0.405
+ (%)	73 (28.7)	39 (39.4)	34 (21.9)	--	SIG =0.001	40 (29.6)	20 (46.5)	20 (21.7)	33 (27.7)	19 (33.9)	14 (22.2)	NS 0.370	NS 0.104	NS 0.472
Fish IgG_4 AbR (kUA/l)	5.53±18.71	10.09±24.69	2.62±12.86	0.01-100.00	SIG <0.001	6.88±21.40	12.95±28.71	4.04±16.38	4.00±15.05	7.89±21.11	0.54±3.13	NS 0.111	NS 0.157	SIG <0.05
+ (%)	28 (11.0)	20 (20.2)	8 (5.2)	--	SIG <0.001	17 (12.6)	10 (23.3)	7 (7.6)	11 (9.2)	10 (17.9)	1 (1.6)	NS 0.199	NS 0.256	SIG <0.05
Meat IgG_4 AbR (kUA/l)	5.41±13.21	7.84±15.82	3.86±11.02	0.04-88.95	SIG <0.01	4.87±12.69	5.84±12.62	4.41±12.76	6.03±13.81	9.38±17.85	3.05±7.82	NS 0.243	NS 0.136	NS 0.225
+ (%)	50 (19.7)	26 (26.3)	24 (15.5)	--	SIG <0.05	26 (19.3)	10 (23.3)	16 (17.4)	24 (20.2)	16 (28.6)	8 (12.7)	NS 0.428	NS 0.278	NS 0.215
Vegetables IgG_4 AbR (kUA/l)	5.74±12.41	7.18±14.28	4.82±11.00	0.01-95.51	NS 0.070	6.35±14.11	7.54±16.25	5.79±13.05	5.06±10.17	6.90±12.71	3.41±6.90	NS 0.205	NS 0.413	NS 0.094
+ (%)	81 (31.9)	37 (37.4)	44 (28.4)	--	NS 0.067	45 (33.3)	15 (34.9)	30 (32.6)	36 (30.3)	22 (39.3)	14 (22.2)	NS 0.300	NS 0.329	NS 0.080
Shellfish/ mollusks IgG_4 AbR (kUA/l)	0.95±4.06	1.06±3.75	0.89±4.25	0.04-34.51	NS 0.370	1.01±4.36	1.46±5.17	0.80±3.94	0.89±3.69	0.75±2.11	1.00±4.69	NS 0.402	NS 0.178	NS 0.387
+ (%)	12 (4.7)	7 (7.1)	5 (3.2)	--	NS 0.080	6 (4.4)	3 (7.0)	3 (3.3)	6 (5.0)	4 (7.1)	2 (3.2)	NS 0.412	NS 0.487	NS 0.488
Milk IgG_4 AbR (kUA/l)	24.95±36.38	27.00±37.01	23.63±36.03	0.00-100.00	NS 0.236	27.18±36.41	29.77±35.79	25.97±36.82	22.41±36.34	24.88±38.10	20.22±34.85	NS 0.149	NS 0.259	NS 0.165
+ (%)	111 (43.7)	45 (45.5)	66 (42.6)	--	NS 0.327	67 (49.6)	23 (53.5)	44 (47.8)	44 (37.0)	22 (39.3)	22 (34.9)	SIG <0.05	NS 0.081	NS 0.056
Fruits IgG_4 AbR (kUA/l)	7.30±11.53	8.02±13.13	6.84±10.40	0.10-23.94	NS 0.213	8.44±12.41	8.66±12.95	8.34±12.22	6.00±10.34	7.54±13.37	4.64±6.41	NS 0.092	NS 0.338	SIG <0.05
+ (%)	107 (42.1)	43 (43.4)	64 (41.3)	--	NS 0.368	61 (45.2)	21 (48.8)	40 (43.5)	46 (38.7)	22 (39.3)	24 (38.1)	NS 0.147	NS 0.173	NS 0.253
Egg IgG_4 AbR (kUA/l)	39.93±39.64	41.21±41.82	39.12±38.29	0.00-64.44	NS 0.342	42.91±40.14	44.18±42.31	42.32±39.31	36.55±38.95	38.92±41.69	34.45±36.55	NS 0.101	NS 0.269	NS 0.105
+ (%)	160 (63.0)	61 (61.6)	99 (63.9)	--	NS 0.359	89 (65.9)	28 (65.1)	61 (66.3)	71 (59.7)	33 (58.9)	38 (60.3)	NS 0.152	NS 0.268	NS 0.225

[a] $sIgG_{4\ CALCULATED}$: mean serum IgG_4 AbR. [b] (+) Percentage of positive food-specific serum ($sIgG_4$) antibody reactions (≥3.5kUA/l).
AbR: antibody reactions.

Table S3. Summary of adverse reactions to food types by sex and age

	M±SD or *n* (%)												
					≤45y (*n*=80)				>45y (*n*=62)				*p*-values$_{age}$
	Total (*n*=142)	Men (*n*=52)	Women (*n*=90)	*p*-values$_{sex}$	Total ≤45y	Men (*n*=28)	Women (*n*=52)	*p*-values$_{sex}$	Total >45y	Men (*n*=24)	Women (*n*=38)	*p*-values$_{sex}$	
**Immune-mediated** [a]													
Total positive sIgG$_4$ AbR	104 (73.2)	37 (71.2)	67 (74.4)	NS 0.671	62 (77.5)	21 (75.0)	41 (78.8)	NS 0.696	42 (67.7)	16 (66.7)	26 (68.4)	NS 0.886	NS 0.194
Total positive sIgE AbR	56 (39.4)	19 (36.5)	37 (41.1)	NS 0.592	34 (42.5)	10 (35.7)	24 (46.2)	NS 0.371	22 (35.5)	9 (37.5)	13 (34.2)	NS 0.794	NS 0.398
Both sIgE + sIgG$_4$ AbR positive	40 (28.2)	10 (19.2)	30 (33.3)	NS 0.073	26 (32.5)	9 (32.1)	17 (32.7)	NS 0.960	14 (22.6)	1 (4.2)	13 (34.2)	SIG <0.01	NS 0.194
positive sIgG$_4$ AbR-only	70 (49.3)	25 (48.1)	45 (50.0)	NS 0.826	43 (53.8)	14 (50.0)	29 (55.8)	NS 0.624	27 (43.5)	11 (45.8)	16 (42.1)	NS 0.775	NS 0.229
positive sIgE AbR-only	11 (7.7)	5 (9.6)	6 (6.7)	NS 0.528	5 (6.3)	1 (3.6)	4 (7.7)	NS 0.470	6 (9.7)	4 (16.7)	2 (5.3)	NS 0.142	NS 0.450
**Non-immune-mediated** [b]													
Positive Lactose intolerance	45 (31.7)	13 (25.0)	32 (35.6)	NS 0.194	24 (30.0)	9 (32.1)	15 (28.8)	NS 0.760	21 (33.9)	4 (16.7)	17 (44.7)	SIG <0.05	NS 0.624
Positive Fructose intolerance	49 (34.5)	18 (34.6)	31 (34.4)	NS 0.984	26 (32.5)	8 (28.6)	18 (34.6)	NS 0.584	23 (37.1)	10 (41.7)	13 (34.2)	NS 0.557	NS 0.569
Both LCI+FCI positive	16 (11.3)	5 (9.6)	11 (12.2)	NS 0.637	8 (10.0)	3 (10.7)	5 (9.6)	NS 0.877	8 (12.9)	2 (8.3)	6 (15.8)	NS 0.397	NS 0.589
LCI-only	29 (20.4)	8 (15.4)	21 (23.3)	NS 0.259	16 (20.0)	6 (21.4)	10 (19.2)	NS 0.816	13 (21.0)	2 (8.3)	11 (28.9)	NS 0.054	NS 0.888
FCI-only	33 (23.2)	13 (25.0)	20 (22.2)	NS 0.707	18 (22.5)	5 (17.9)	13 (25.0)	NS 0.468	15 (24.2)	8 (33.3)	18.4 (7.0)	NS 0.185	NS 0.813

[a] Positive immune-mediated reactions: ≥3.5 kUA/l.
[b] Positive non-immune-mediated reactions: Hydrogen H_2≥20ppm and/or methane CH_4≥12ppm.
LCI, lactose intolerance; *FCI*, fructose intolerance; *sIgE*, serum immunoglobulin E; *sIgG$_4$*, immunoglobulin G_4.

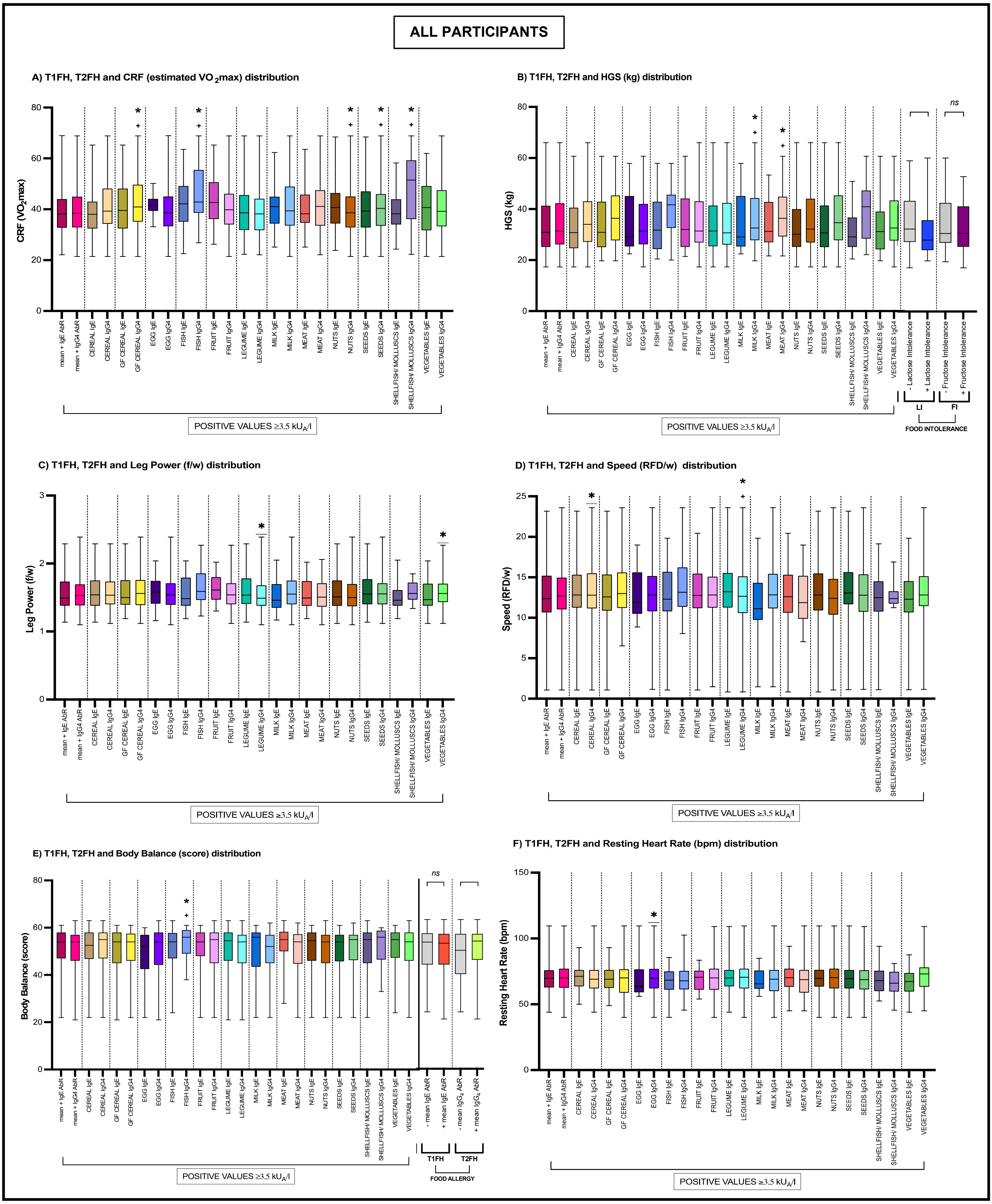

Figure S1. Physical Performance variables and adverse reactions to foodstuff against 12 food groups of the total studied sample of Spanish adults. *$p < 0.05$, **$p < 0.01$, ***$p < 0.001$, ****$p < 0.0001$. +, positive association; -, negative association; bpm, beats per minute; CRF, cardiorespiratory fitness; f/w, force/weight; GF, gluten-free; HGS, handgrip strength; IgE, immunoglobulin E; IgG_4, immunoglobulin G_4; T1FH, type 1 food hypersensitivity; T2FH, type 2 food hypersensitivity; VO_2max, maximal oxygen consumption.

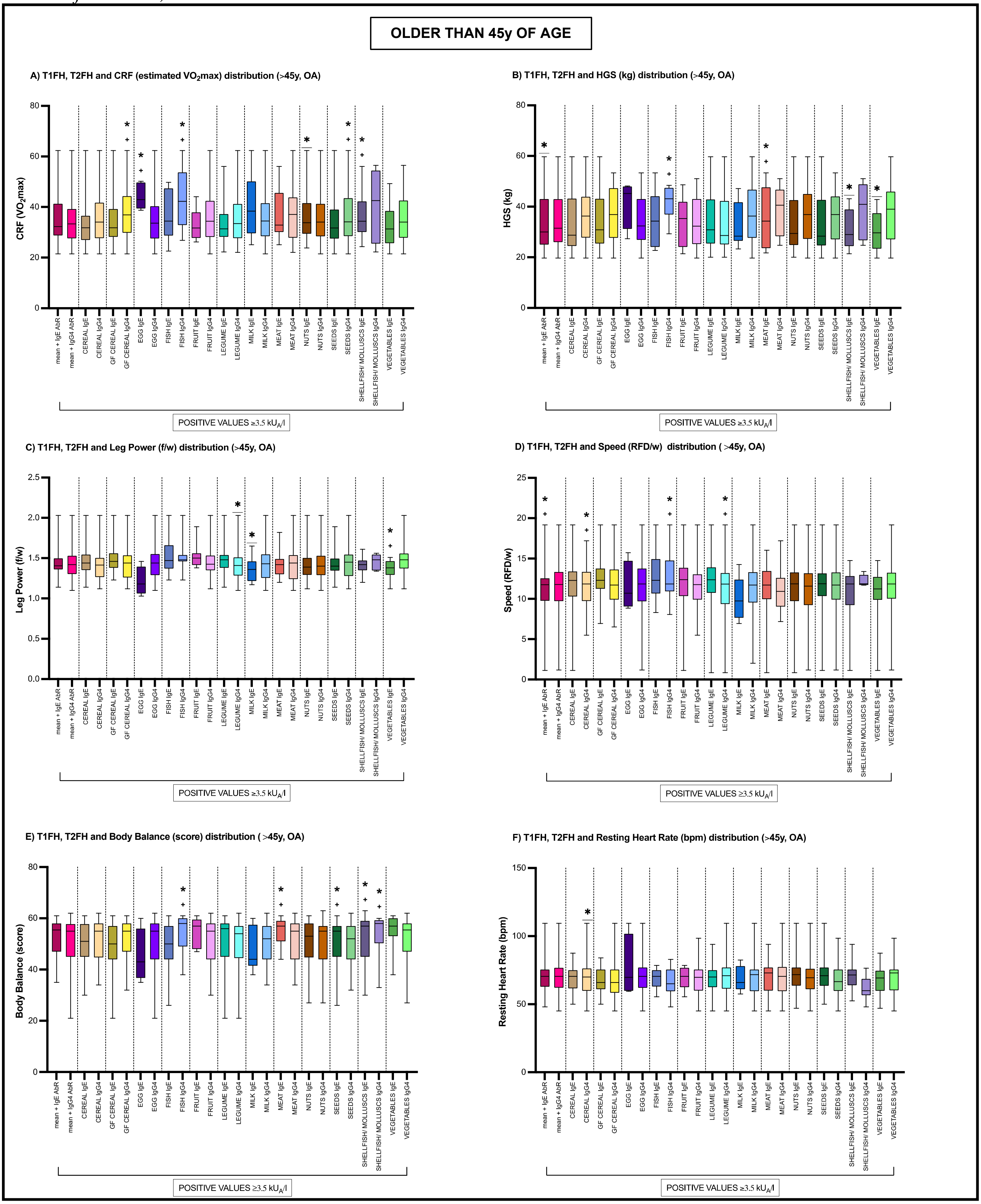

Figure S2. Physical Performance variables and adverse reactions to foodstuff against 12 food groups of Spanish adults older than 45y of age. *p< 0.05, **p< 0.01, ***p< 0.001, ****p< 0.0001. +, positive association; -, negative association; bpm, beats per minute; CRF, cardiorespiratory fitness; f/w, force/weight; GF, gluten-free; HGS, handgrip strength; IgE, immunoglobulin E; IgG_4, immunoglobulin G_4; T1FH, type 1 food hypersensitivity; T2FH, type 2 food hypersensitivity; VO_2max, maximal oxygen consumption.
